# Molecular Imaging of Human Skeletal Myoblasts (huSKM) in Mouse Post-Infarction Myocardium

**DOI:** 10.3390/ijms221910885

**Published:** 2021-10-08

**Authors:** Katarzyna Fiedorowicz, Weronika Wargocka-Matuszewska, Karolina A. Ambrożkiewicz, Anna Rugowska, Łukasz Cheda, Michał Fiedorowicz, Agnieszka Zimna, Monika Drabik, Szymon Borkowski, Maciej Świątkiewicz, Piotr Bogorodzki, Paweł Grieb, Paulina Hamankiewicz, Tomasz J. Kolanowski, Natalia Rozwadowska, Urszula Kozłowska, Aleksandra Klimczak, Jerzy Kolasiński, Zbigniew Rogulski, Maciej Kurpisz

**Affiliations:** 1 Institute of Human Genetics, Polish Academy of Sciences, 60-479 Poznan, Poland; katarzyna.fiedorowicz@igcz.poznan.pl (K.F.); karolina.bednarowicz@igcz.poznan.pl (K.A.A.); agnieszka.zimna@igcz.poznan.pl (A.Z.); Tomasz.kolanowski@igcz.poznan.pl (T.J.K.); natalia.rozwadowska@igcz.poznan.pl (N.R.); k.szurula@gmail.com (U.K.); aleksandra.klimczak@hirszfeld.pl (A.K.); 2 Biological and Chemical Research Centre, Faculty of Chemistry, University of Warsaw, 02-089 Warsaw, Poland; wargocka.w@gmail.com (W.W.-M.); lcheda@chem.uw.edu.pl (Ł.C.); phamankiewicz@cnbc.uw.edu.pl (P.H.); rogul@chem.uw.edu.pl (Z.R.); 3 Faculty of Biology, Institute of Human Biology and Evolution, Adam Mickiewicz University, 60-479 Poznan, Poland; anna.rugowska@amu.edu.pl; 4 Mossakowski Medical Research Institute, Polish Academy of Sciences, 02-106 Warsaw, Poland; mfiedorowicz@imdik.pan.pl (M.F.); monika.angelika.drabik@gmail.com (M.D.); swiatkiewicz.maciej@gmail.com (M.Ś.); p.bogorodzki@ire.pw.edu.pl (P.B.); pgrieb@imdik.pan.pl (P.G.); 5 Institute of Radioelectronics and Multimedia Technology, Warsaw University of Technology, 00-665 Warsaw, Poland; borekszymon94@gmail.com; 6 Institute of Bioorganic Chemistry, Polish Academy of Sciences, 61-704 Poznan, Poland; 7 Hirszfeld Institute of Immunology and Experimental Therapy, Polish Academy of Sciences, 53-114 Wroclaw, Poland; 8 Kolasiński Clinic, Hair Clinic Poznan, 62-020 Swarzędz, Poland; colas@klinikakolasinski.pl

**Keywords:** human skeletal myoblasts, mesenchymal stem cells, Single-Photon Emission Computed Tomography/Computed Tomography, Magnetic Resonance Imaging, Bioluminescent Imaging, promoter reporter gene, technetium

## Abstract

Current treatment protocols for myocardial infarction improve the outcome of disease to some extent but do not provide the clue for full regeneration of the heart tissues. An increasing body of evidence has shown that transplantation of cells may lead to some organ recovery. However, the optimal stem cell population has not been yet identified. We would like to propose a novel pro-regenerative treatment for post-infarction heart based on the combination of human skeletal myoblasts (huSkM) and mesenchymal stem cells (MSCs). huSkM native or overexpressing gene coding for Cx43 (huSKMCx43) alone or combined with MSCs were delivered in four cellular therapeutic variants into the healthy and post-infarction heart of mice while using molecular reporter probes. Single-Photon Emission Computed Tomography/Computed Tomography (SPECT/CT) performed right after cell delivery and 24 h later revealed a trend towards an increase in the isotopic uptake in the post-infarction group of animals treated by a combination of huSkMCx43 with MSC. Bioluminescent imaging (BLI) showed the highest increase in firefly luciferase (fluc) signal intensity in post-infarction heart treated with combination of huSkM and MSCs vs. huSkM alone (*p* < 0.0001). In healthy myocardium, however, nanoluciferase signal (nanoluc) intensity varied markedly between animals treated with stem cell populations either alone or in combinations with the tendency to be simply decreased. Therefore, our observations seem to show that MSCs supported viability, engraftment, and even proliferation of huSkM in the post-infarction heart.

## 1. Introduction

Myocardial infarction (MI) is a leading cause of morbidity and mortality worldwide. Pharmacological and surgical treatments improve the outcome of this disease to some extent; however, known conventional therapies do not provide the regeneration or replacement of the damaged heart tissues. Over the last decade, an increasing body of evidence has shown that transplantation of stem/progenitor cells may lead to promising recovery of cardiac function [1]. Even though various types of stem/progenitor cells (bone marrow stem cells, skeletal myoblasts (huSkM), mesenchymal stem cells (MSCs), embryonic stem cells, or iPSC-derived cardiomyocytes) have been proposed and tested [2], the optimal cell population has not been identified. The major reason for the poor effects of so far performed cellular therapies was linked to limitations such as cell migration from the target site of their administration, insufficient trans-differentiation or differentiation into adult cardiomyocytes, induction of an arrhythmia, or tumour formation [3]. Therefore, we suggest that combined cellular therapy using huSkM and MSC should be pursued in order to use the “best features” of a particular cell type.

huSkM represent one of the best studied (in in vivo animal models [4,5] and in humans [6,7]) cell candidates to support heart regeneration after MI since huSkM can be easily isolated and propagated in in vitro culture. These cells are also resistant to hypoxic conditions prevailing in post-infarction myocardium [8]. Previous reports have shown that myoblast therapy improves the functionality of the MI heart, affecting angiogenesis [9,10], inflammation [11], matrix remodelling, tissue cell reservoir proliferation (CPC-cardiac progenitor cells), or inhibition of cardiomyocytes apoptosis. Unfortunately, huSkM do not express connexin 43 (Cx43)–the principal element of functional gap junctions that is critical for cell-to-cell communications [12]. We [13] and others have previously shown that introducing the Cx43-overexpressing vectors led to the improvement of electrical coupling in vitro [14,15] and in vivo between neighbouring cardiomyocytes localised in the peri-infarction zone and the surrounding myocardium [16].

Mesenchymal stem cells (MSC) are another type of cells used in post-infarction heart therapy. Their regenerative potential, to a large extent, relies on the ability to secrete bioactive factors at variable concentrations induced in response to local microenvironmental cues [17,18]. The studies of Kinnaird et al. [19] and Haynesworth et al. [20] showed that released basic fibroblast growth factor (bFGF) and granulocyte colony-stimulating factor (G-CSF) induced the proliferation of endothelial cells, smooth muscle cells, and neutrophils, respectively. In turn, the release of insulin like growth factor (IGF), epidermal growth factor (EGF) and vascular endothelial growth (VEGF) enhanced the angiogenesis. Mesenchymal stem cells modulate the local immune response and may thus establish a favourable milieu of complementary candidates for a pro-regenerative microenvironment (niche) in the target organ.

Here, we propose a novel pro-regenerative treatment for the post-infarction heart based on the features of huSkM and MSC cells. Since determination of cell homing (retention), distribution and engraftment are the initial prerequisites for successful cell therapy; thus, we proposed to implement a combination of modern molecular imaging techniques to gain detailed information on the distribution, viability and proliferation of stem cells applied in the proposed model of cellular therapy. In our studies, we used Single-photon emission computed tomography (SPECT) with technetium-99m (^99m^Tc) as a radiotracer. This allowed for short-term but sensitive determination of the efficiency of cell delivery. For long-term follow-up, Bioluminescent imaging (BLI) was used. This noninvasive in vivo cell tracking is based on the fluorescent or luminescent signals generated by an inserted reporter gene promoter, whose expression corresponds to the number of viable cells. Finally, magnetic resonance imaging (MRI) was performed to assess the success of the proposed treatment.

## 2. Results

### 2.1. Experimental Design

**Figure 1:**
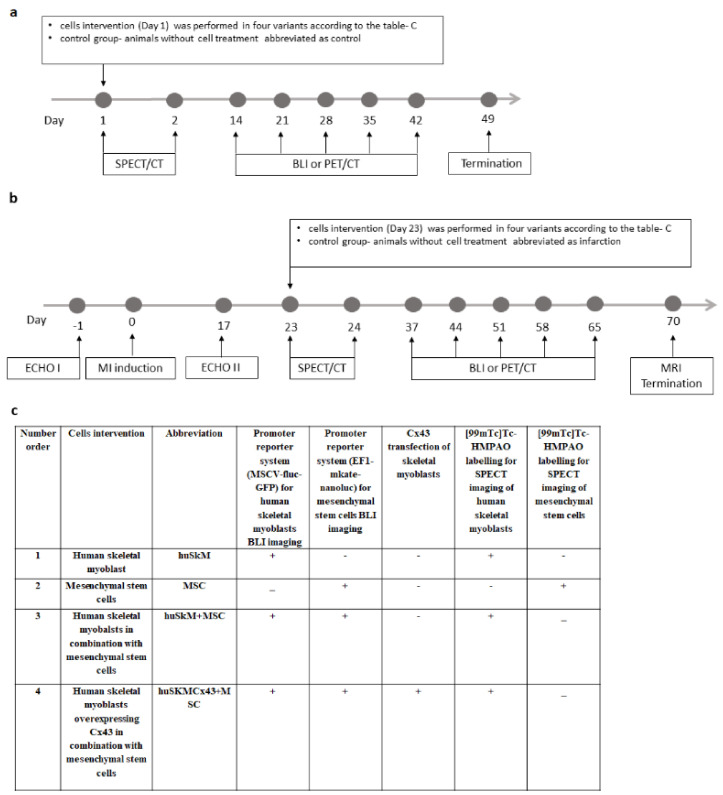
Scheme of experimental design of in vivo animal experiments. (**a**). Procedure 1 was performed in NOD/SCID mice with the healthy heart. On day 0, immunocompromised mice were divided into 4 subgroups based on the cell intervention. Four different combinations of cells were transplanted into the healthy mouse heart at day 1. 1. huSkM- human skeletal myoblasts, 2. MSC- mesenchymal stem cells 3. huSkM + MSC- human skeletal myoblasts in combination with mesenchymal stem cells, 4. huSkMCx43+MSC- human skeletal myoblasts overexpressing Cx43 in combination with mesenchymal stem cells. SPECT/CT was performed immediately after the transplantation and 24 h later Then, the biodistribution of transplanted cells was observed using a bioluminescent imaging system (BLI) for 6 weeks (day 14,21,28,35,42). On day 49, the animals were terminated. (**b**). According to Procedure 2, initial control echocardiography (ECHO I) was performed prior to the in vivo experimental series, and myocardial infarction (MI) was induced by left coronary artery ligation on day 0. Seventeen days later, a second echocardiography (ECHO II) confirming successful MI induction was performed. On day 23, cell intervention was performed in the same four combinations as described for Procedure 1. SPECT/CT was performed immediately after the transplantation and 24 h later. The retention of the cells in the myocardium was observed using a BLI system up to 10 weeks (day 37, 44,51,58,65). On day 70, MRI imaging was performed, and the animals were terminated. Untreated animals (infarction) were used as the controls, and PET/CT and MRI were performed in this group for comparison. (**c**). Table indicates stem cells modifications that were introduced prior to their in vivo transplantation. In order to image huSkM and MSCs concomitantly using BLI, each cell type was transduced with different lentiviral promoter reporter systems. huSkM cells were transduced with MSCV-fluc-GFP, where constitutive expression of MSCV promoter controls the expression of firefly luciferase (fluc) and green fluorescent protein (GFP). MSC cells were transduced with EF1-mkate-nanoluc, where constitutive expression of EF1 (Elongation Factor1) controls the expression of red fluorescence (mkate) and nanoluciferase (nanoluc). In one of the transplanted cells combinations (huSkMCx43+MSC), in order to enhance expression of connexin 43, huSkM cells were transfected with pCiNeo plasmid containing sequence for Cx43 by electroporation. Finally, to image huSkM cells (in all transplant variants) and MSC (when transplanted alone) right after delivery (and 24 h later) using SPECT/CT technique, cells were labelled with [^99m^Tc]Tc-HMPAO.

In vivo experiments were divided into two procedures. In Procedure 1 (Figure 1a), 4 different combinations of previously characterised cells (out of passage 6) were transplanted into healthy animal hearts on day 1: 1. huSkM- human skeletal myoblasts, 2. MSC- mesenchymal stem cells 3. huSkM + MSC–human skeletal myoblasts in combination with mesenchymal stem cells, 4. huSkMCx43 + MSC = human skeletal myoblasts overexpressing Cx43 in combination with mesenchymal stem cells. SPET/CT was performed immediately after cell administration and 24 h later (day 1st and 2nd). Then, cells biodistribution was observed using an in vivo imaging system (BLI) for 6 weeks (day 14,21,28,35,42). On day 49, the animals were terminated.

In Procedure 2 (Figure 1b), the first control animal echocardiography (ECHO I) was performed prior to the experimental series, and myocardial infarction (MI) was induced by left coronary artery ligation on day 0. In this case, 17 days later, a second echocardiography (ECHO II) confirming successful MI induction was performed. On day 23, the cell intervention was performed in the same four variants as described for Procedure 1. SPECT/CT was performed immediately after transplantation and 24 h later. The retention of the cells in the myocardium was observed using a BLI system of bioluminescence for up to 10 weeks (day 37,44,51,58,65). On day 70, MRI imaging was performed, and the animals were terminated. Control and post-infarction hearts (without cell intervention, administered with 0.9% NaCl solution) were used as the corresponding controls.

To be able to image two types of transplanted cells using concomitant BLI, each cell type was transduced with different lentiviral promoter reporter systems. huSkM cells were transduced with murine stem cell virus, MSCV-fluc-GFP, where constitutive expression of MSCV promoter controls the expression of firefly luciferase (fluc) and green fluorescent protein (GFP). MSC cells were transduced with EF1-mkate-nanoluc, where constitutive expression of EF1 (Elongation Factor1) controls the expression of red fluorescence (mkate) and nanoluciferase (nanoluc). In one of the transplanted cells combinations (huSkMCx43 + MSC), in order to enhance expression of connexin 43, huSkM cells were transfected with pCiNeo plasmid containing sequence for Cx43 by electroporation. Finally, to image huSkM cells (in all transplant variants) and MSC (when delivered alone) right after delivery (and 24 h later) using SPECT/CT technique, cells were labelled with Technetium-99m Hexamethylpropyleneamine Oxime ([^99m^Tc]Tc-HMPAO) (Figure 1c). To make the study easier to follow the abbreviations used throughout the text and figures always refer to the cells that were indicated in Figure 1c.

### 2.2. Characteristics of huSkM 

**Figure 2:**
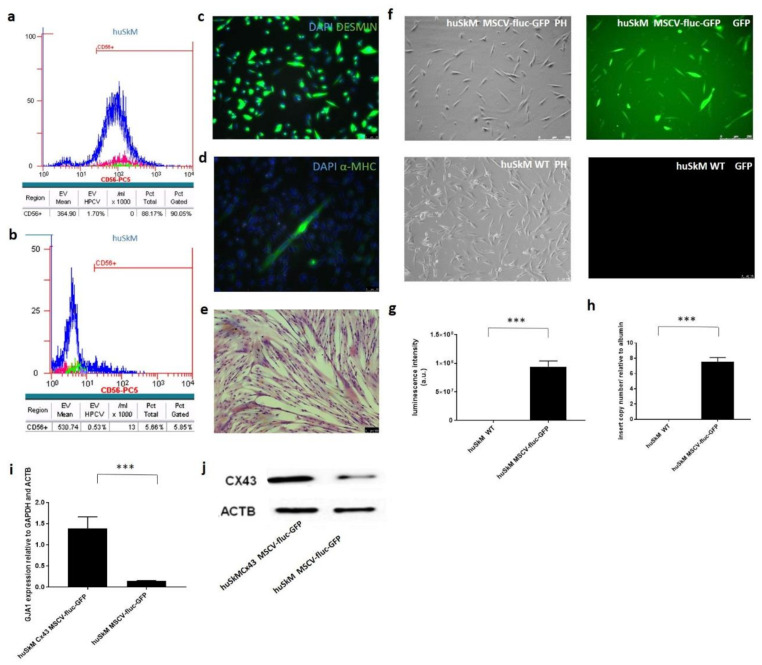
Characteristics of huSkM and evaluation of the efficiency of huSkM transduction with MSCV-fluc-GFP reporter vector. (**a**). Flow cytometry detected approximately 90% of CD56^+^ huSkM cells in the isolated skeletal muscle population. (**b**). Isotype control was performed in parallel (IgG1-PC5). (**c**). Immunofluorescence of huSkM stained with an anti-desmin antibody (green) and nuclear dye DAPI (blue), scale bar = 50 μm. (**d**). Immunofluorescence of huSkM stained with an anti-α-MHC (myosin heavy chain) antibody (green) and nuclear dye DAPI (blue), scale bar = 50 μm. (**e**). Multinuclear tube formation test confirmed the ability of the cells to differentiate in vitro, scale bar = 50 μm. (**f**). huSkM transduced with MSCV-fluc-GFP-lentiviral reporter vector vs. negative control cells. (**g**). Firefly luciferase luminescence activity measured in transduced cells (huSkM MSCV-fluc-GFP) vs. negative control cells (huSkM WT) in triplicate, *** *p* > 0.001. (**h**). MSCV-GFP-luc insert copy number in huSkM cells (7.5 copies) vs. untransduced control (huSkM WT) relative to albumin, *** *p* < 0.003. (**i**). Relative expression of the gene encoding for Cx43 in pCiNeo-CX43-transfected huSkM 48 h after the transfection vs. untransfected cells, *** *p* < 0.001. (**j**). Overexpression of Cx43 protein in pCiNeo-CX43-transfected huSkM vs. untransfected cells 48 h after the transfection. ACTB was used as a loading control.

Flow cytometry analysis of myogenic marker (CD56) revealed approximately 90% of positive cells (Figure 2a). The isotypic negative control has been shown in Figure 2b. High expression of desmin and low αMHC expression (marker of differentiated myogenic cells) were confirmed by immunofluorescence, as shown in Figure 2c,d. The myotube formation test showed that the characterised cells retained their function in vitro (Figure 2e). To establish a promoter reporter cell line, huSkM were transduced with MSCV-fluc-GFP-puromycin lentiviral particles. The GFP–positive signal driven by the constitutive MSCV promoter was imaged using a fluorescence microscope and compared to the signal of untransduced cells (Figure 2f). A significant increase in firefly luciferase expression in in vitro cell culture in transduced vs. untransduced cells is shown in Figure 2g. The insert copy number was estimated using quantitative polymerase chain reaction (q-PCR) to be about 7 copies of MSCV-fluc-GFP reporter vector inserted into the transduced huSkM population (Figure 2h). The established cell line was then transfected with the pCiNeo plasmid containing sequence for Cx43 by electroporation. In this case, 48 h after the transfection, significant overexpression of Cx43 was confirmed by real-time PCR (Figure 2i) and Western blotting, as shown in Figure 2j.

### 2.3. Characteristics of Mesenchymal Stem Cells 

**Figure 3:**
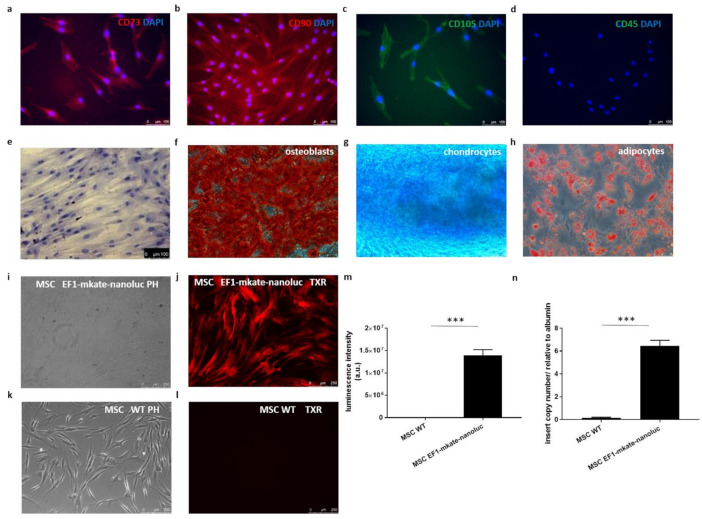
Mesenchymal stem cells characteristics. (**a**). Cells stained positively for-CD73, (**b**). CD90 (**c**). CD 105 and (**d**). negatively for CD 45, nuclear dye DAPI (blue), scale bar = 100 μm. (**e**). Staining of MSC with eosin and hematoxylin. Multipotent character of the cells shown as the ability to differentiate into osteoblasts, chondrocytes and adipocytes was confirmed with (**f**). Alcian red, (**g**). Alizarin blue and (**h**). Oil red staining, respectively, scale bar = 50 μm. (**i**,**j**). in vitro imaging of mesenchymal cells transduced with EF-1-mkate-nanoluc lentiviral promoter reporter system vs. (**k**,**l**). Non transduced cells–WT. (**m**). Luminescence intensity of EF-1-mkate-nanoluc-transduced mesenchymal stem cells vs. untransduced controls (WT); measurements were performed in triplicate, *** *p* < 0.001. (**n**). EF1-mkate-nanoluc insert copy number in MSC cells (6.4 copies) vs. untransduced control (MSC WT) relative to albumin, *** *p* < 0.0001.

Mesenchymal stem cells phenotype was characterised by immunofluorescence and flow cytometry. As shown in Figure 3, cells were positive for CD 73 (a), CD90 (b), CD 105 markers (c) and negative for CD45 marker (d). Markers expression positive for CD 73 (Supplementary Figure S1a) and CD90 (Supplementary Figure S1b), while negative for CD34 (Supplementary Figure S1c), and CD45 (Supplementary Figure S1d) have been placed in Supplemental File. Adipose tissue derived mesenchymal stem cells staining for eosin and hematoxylin were shown in Figure 3e. The cells were able to differentiate into adipocytes, chondrocytes and osteoblasts, as confirmed by Oil Red O, Alcian blue and Alizarin red staining, respectively (Figure 3f–h). Phenotypically and functionally characterised cells were then transduced with EF-1-mkate-nanoluc lentiviral particles. The positive mkate signal was imaged using a fluorescence microscope and compared to that one in untransduced control cells (Figure 3i–l). A significant increase in the intensity of the bioluminescent signal in transduced vs. untransduced cells was measured using a luminometer, as demonstrated in Figure 3m. As shown in Figure 3n, the average copy number of the EF-1-mkate-nanoluc insert was 6.4 according to the qPCR data.

### 2.4. Echocardiography and PET/CT

**Figure 4:**
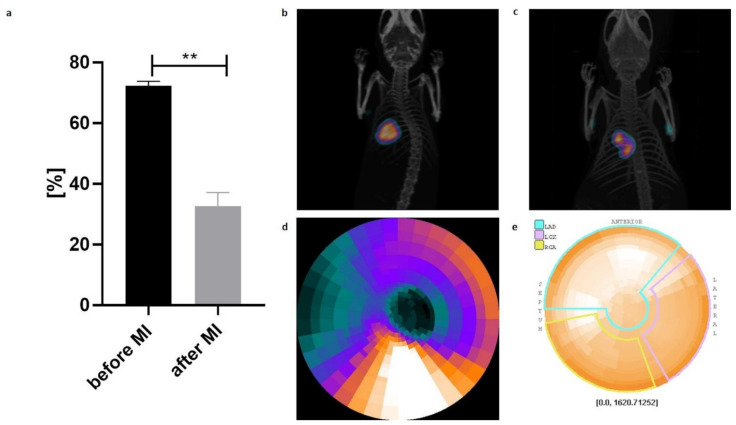
Haemodynamic parameters of cardiac function with respect to induced myocardial infarction (MI). (**a**) Echocardiography performed 17 days after MI induction revealed a significant decrease in % SAX (area of change in short axis) compared to that in the control mice prior to MI (*n* = 40, data are presented as the mean ± SD, ** *p* < 0.01, *t*-student test); (**b**) Representative images of [^18^F]-FDG PET/CT scan of a control normal heart; (**c**) Representative images of [18F]-FDG PET/CT scan of post-infarction heart; (**d**) Conversion of PET images to maps of radiotracer activity in the MI heart and their (**e**) counterparts presented as polar maps.

An echocardiography procedure was performed to confirm successful MI induction. MI induction led to a significant decrease in the ejection fraction (Figure 4a). Positron emission tomography/computed tomography (PET/CT) imaging revealed altered 2-deoxy-18F-fluorodeoxyglucose ([^18^F]-FDG) tracer uptake in the MI group (Figure 4b,c). Next, PET images were converted to maps of radiotracer activity in the MI heart (d) and their counterparts were presented as polar maps (e). 

### 2.5. SPECT/CT

**Figure 5:**
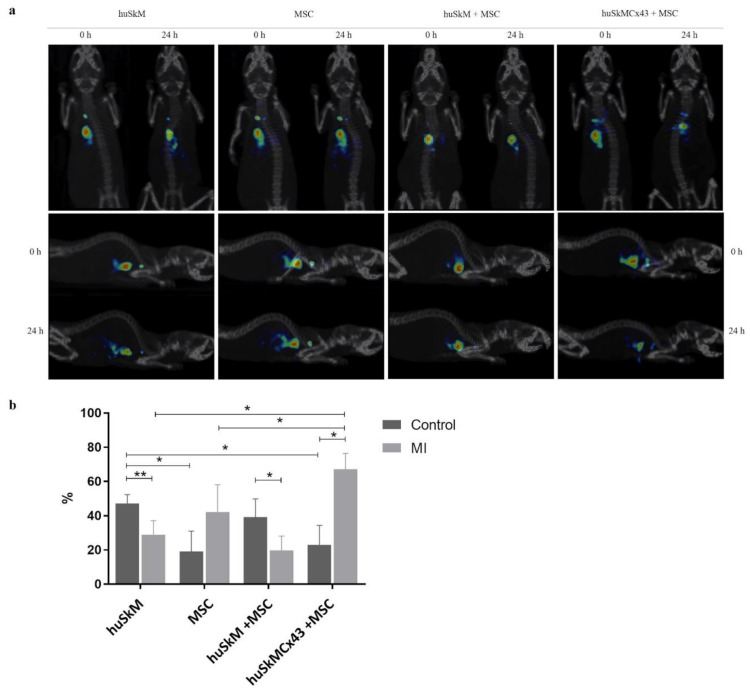
(**a**). 3D SPECT/CT images of various cell combinations labelled with [^99m^Tc]Tc-HMPAO administered intramyocardially. Images were acquired at 0 min and after 23 ± 1 h. (**b**). The efficiency of the colonization of stem cells (huSkM, MSC, huSkM + MSC, huSkMCx43 + MSC) labelled with [^99m^Tc]Tc-HMPAO at 24 h in post-infarction mice (healthy hearts: huSkM *n* = 7, MSC *n* = 8, huSkM + MSC *n* = 4, huSkMCx43+MSC *n* = 4; post-infarction hearts huSkM *n* = 8, MSC *n* = 3, huSkM + MSC *n* = 8, huSkMCx43+MSC *n* = 8). Data are presented as the mean ± SD (* *p* < 0.05, Mann-Whitney U test).

The mean cell labelling efficiency was 48.1 ± 22.7%. A 20 min image acquisition started immediately after the intramyocardial injection of [^99m^Tc]Tc-HMPAO-labelled stem cells, and images were acquired by single-photon emission computed tomography/computed tomography (SPECT/CT). A second late planar image was obtained from 21 to 24 h after cell administration. Images shown in Figure 5a were obtained from the representative post-infarction mice. The results were corrected for the decay of [^99m^Tc]Tc-HMPAO and were expressed as the percentage change based on the values obtained in all groups of mice as a ratio of standardized uptake value (SUV) of the first measurement to SUV of the second measurement (mean ± SD). Cell colonisation in the healthy heart of control mice was the highest in huSkM (although not significant); while in the post-infarction mice, the cell colonisation was the highest in huSkMCx43+MSC combination (Figure 5b). Control and post-infarction mice were then compared, and a trend towards an increase in the isotopic uptake due to cell intervention in the post-infarction heart was clearly observed in huSkMCx43+MSC (67.17 ± 9.30) compared to the other post-infarction cell interventions used in the study (Supplementary Table S1).

### 2.6. BLI

**Figure 6:**
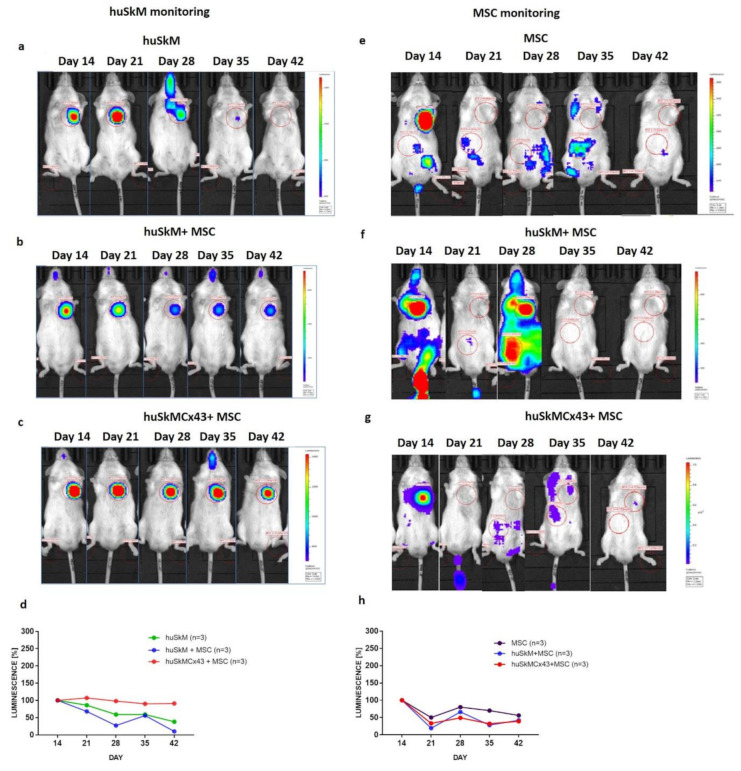
In vivo long-term monitoring of intramyocardially transplanted cells in a healthy mouse model (heart) determined by bioluminescence imaging. Images were taken on days 14, 21, 28, 35 and 42 after their delivery to the normal physiological heart. Each image is displayed on a rainbow scale in units of photon radiance (s^–1^ cm^–2^ sr^–1^) and overlaid onto a greyscale reference image of the corresponding mouse. Representative images of firefly luciferase luminescence intensity of huSkM transduced with MSCV-fluc-GFP reporter vector in (**a**). huSkM, (**b**). huSkM + MSC and (**c**). huSkMCx43 + MSC. (**d**). The percentage (%) of firefly luciferase luminescence signal intensity in huSkM transduced with MSCV-fluc-GFP reporter vector on days 14, 21, 28, 35 and 42 after the intervention (healthy heart) plotted for huSkM (*n* = 3), huSkM + MSC (*n* = 3), and huSkMCx43+MSC (*n* = 3) (one-way Anova test followed by Tukey’s multiple comparison test). Representative images of nanoluciferase luminescence intensity in MSCs transduced with EF-1-mkate-nanoluc reporter vector in (**e**). MSC, (**f**). huSkM + MSC and (**g**). huSkMCx43 + MSC. (**h**) The percentage (%) of nanoluciferase signal intensity in MSC EF1-mkate-nanoluc reporter vector on days 14, 21, 28, 35 and 42 after the intervention (healthy heart) plotted for MSC, huSkM + MSC, and huSkMCx43 + MSC (one-way Anova test followed by Tukey’s multiple comparison test).

**Figure 7:**
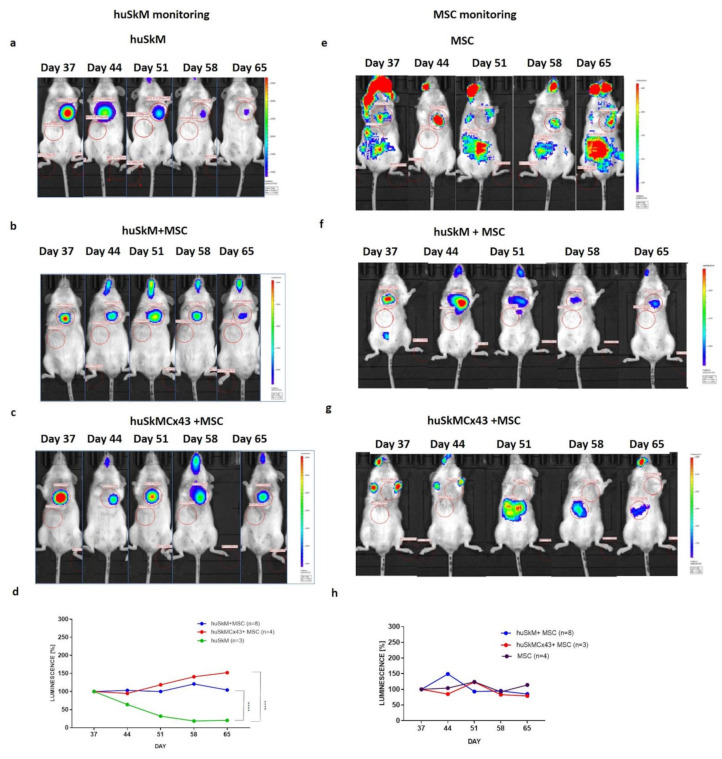
In vivo long-term monitoring of stem cells intramyocardially transplanted into a post-infarction mouse heart determined by bioluminescence imaging. Images were acquired on days 37, 44, 51, 58 and 65 of the experiment. Each image is displayed on a rainbow scale in units of photon radiance (s^–1^ cm^–2^ sr^–1^) and overlaid onto a greyscale reference image of the corresponding mouse. Representative images of firefly luciferase luminescence intensity in (**a**). huSkM, (**b**). huSkM + MSC and (**c**). huSkMCx43 + MSC. (**d**). The percentage (%) of firefly luciferase luminescence signal intensity in huSkM transduced with MSCV-fluc-GFP reporter vector on days 37, 44, 51, 58 and 65 of the experiment (post-infarction heart) plotted for huSkM (*n* = 3), huSkM+MSC (*n* = 8) and huSkMCx43+MSC (*n* = 4), (**** *p* < 0.0001, one-way Anova test followed by Tukey’s multiple comparison test). Representative images of nanoluciferase luminescence intensity in MSCs transduced with EF-1-mkate-nanoluc reporter vector in (**e**). MSCs, (**f**). huSkM + MSC and (**g**). huSkMCx43+MSC. (**h**). The percentage (%) of nanoluciferase signal intensity in MSCs transduced with EF1-mkate-nanoluc reporter vector on days 37, 44, 51, 58 and 65 of the experiment (post-infarction hearts) plotted for MSCs (*n* = 3), huSkM+MSC (*n* = 8), and huSkMCx43 + MSC (*n* = 4) (one-way Anova test followed by Tukey’s multiple comparison test).

The biodistribution of transplanted stem cells was monitored using bioluminescence imaging (Figure 6 and Figure 7). In the normal control heart, the firefly luciferase luminescence intensity corresponded to the viability of huSkM and had a tendency to decrease in huSkM alone and huSkM in combination with MSC (Figure 6a,b,d); in huSkMCx43 in combination with MSC the bioluminescent signal remained stable (Figure 6c,d). There were no significant differences in bioluminescence intensity between tested combinations (Figure 6d). Mesenchymal stem cells retention was imaged as the intensity of nanoluciferase in the normal healthy heart. The MSCs signal intensity decreased 21 days post treatment in all tested variants (huSkM, MSC, huSkM + MSC and huSkMCx43 + MSC) and increased again 7 days later (day 28). At the end of monitoring imaging there were no significant differences in luminescence intensity between all tested variants (Figure 6e–h). 

The total firefly luciferase intensity signal characteristic for (huSkM) was significantly increased in the post-infarction hearts of mice treated with various cell combinations. In huSkM+MSC and huSkMCx43+MSC revealed statistical significance versus huSkM alone (*p* < 0.0001) over the five-week imaging period (Figure 7a–d). In the case of mesenchymal stem cells which expressed nanoluciferase, the signal intensity for all tested variants was transiently on increase at day 44 (huSkM + MSC) or at day 51 (MSC alone and huSkMCx43 + MSC) and subsequently decreased to the level detected at day 37. There were no significant differences in the nanoluciferase signal intensity in all the combinations treated for mesenchymal stem cells retention in post-infarction heart.

### 2.7. MRI

**Figure 8:**
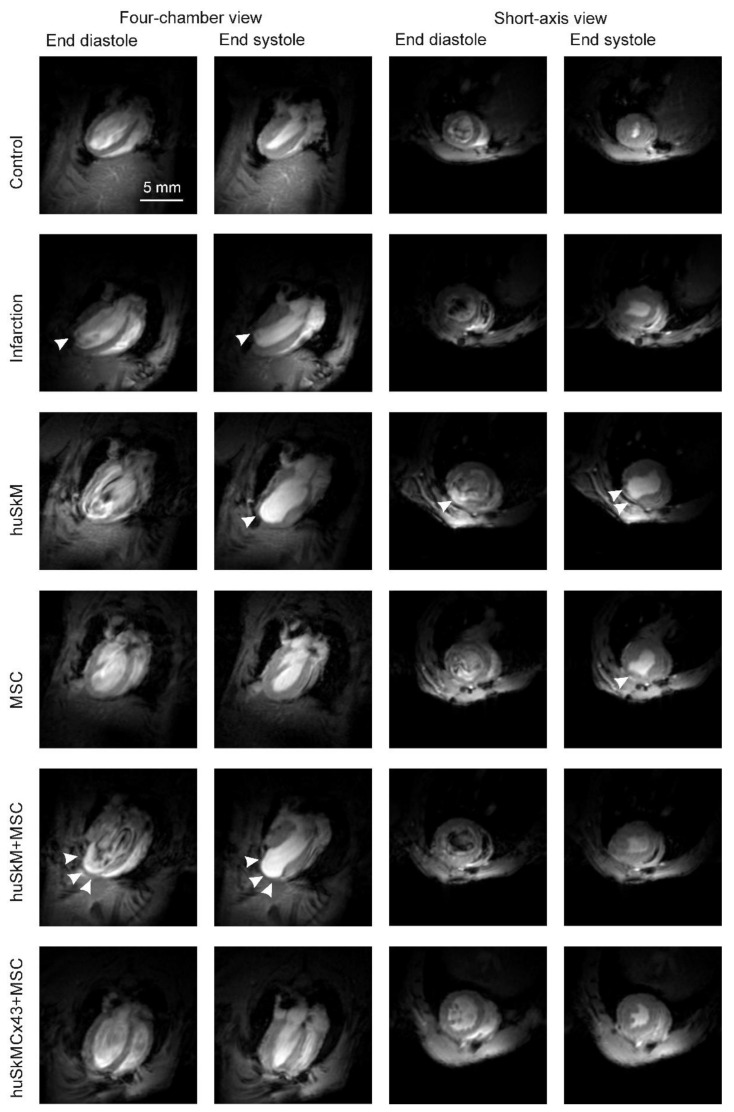
Representative MR images of control hearts (no interventions), post-infarction hearts, and healthy hearts after huSkM, MSCs, huSkM + MSC or huSkMCx43 + MSC cell interventions. The first two columns represent the 4-chamber view (end-diastole is the first column and end-systole is the second column); the next two columns represent the short-axis view (end-diastole is the third column and end-systole is the fourth column). The infarct area is indicated by arrows.

**Figure 9:**
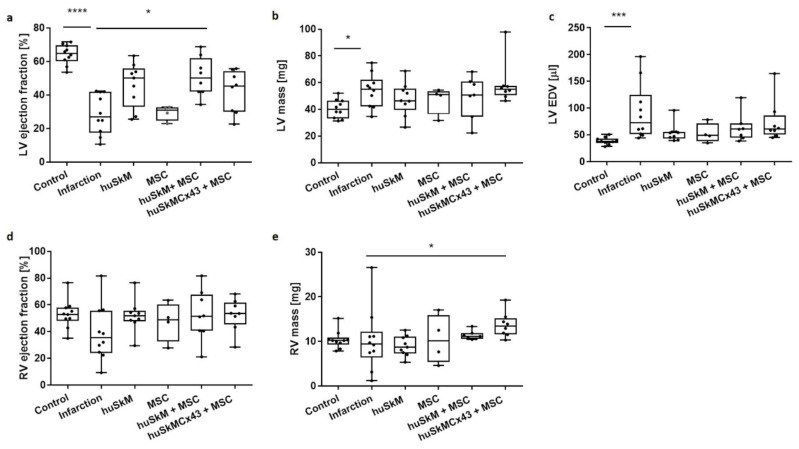
Functional analysis of cardiac haemodynamics after huSkM (*n* = 4), MSCs (*n* = 4), huSkM+MSC (*n* = 8 and huSkMCx43+MSC (*n* = 8) cells intervention compared to the control-healthy hearts (*n* = 10) and MI (*n* = 5). (**a**). left ventricle ejection fraction (LV ejection fraction), (**b**). left ventricle mass (LV mass), (**c**). left ventricle end-diastolic volume (LV EDV), (**d**). right ventricle ejection fraction (RV ejection fraction) and (**e**). right ventricle mass (RV mass). Box represents 25th–75th percentile, line represents the median, whiskers are min to max, single values are presented as dots. * *p* < 0.05, *** *p* < 0.001, **** *p* < 0.0001, Kruskal-Wallis test followed by Dunn’s multiple comparisons test.

Figure 8 presents data from representative functional MRI images of the control (healthy hearts), post-infarction hearts (MI) and post-infarction hearts after the treatment with various combinations of cell interventions: huSkM, MSC, huSkM + MSC and huSkMCx43 + MSC. The calculation of left ventricle ejection fraction (LV-EF) (Figure 9a) revealed a significant decrease after induced MI compared to that one in the healthy controls. Treatment with huSkM alone or in combination with mesenchymal stem cells resulted in an increase in LV-EF; however, the changes reached a statistically significant level (*p* < 0.02) only in the case of cell intervention with huSkM+MSC. LV mass was significantly increased in MI mice compared to that one in the control group (*p* < 0.03). In mice treated with stem cells, LV mass was lower than that one in MI group (Figure 9b). Left ventricular end-diastolic volume (LV-EDV) was significantly increased in the MI group compared to that one in control group (*p* < 0.0002) and did not change significantly in the groups of animals treated with various combinations of stem cells (Figure 9c). Right ventricle ejection fraction (RV-EF) was similar in all combinations of all cell interventions (Figure 9d); however, RV mass was significantly increased in cell intervention of huSkMCx43+MSC (*p* < 0.04) (Figure 9e).

## 3. Discussion

Several studies considered regeneration of the post-infarction heart as the “holy grail”. Positive efforts obtained in preclinical animal models have not yet been positively translated into clinical practice. Successful organ regeneration should involve several crucial steps, starting from identification of an optimal source of the delivered cells (possibly from tissue reservoirs) or their combinations through the proper cell homing (retention) in target organ, long-term cell engraftment and then recovery of function that leads to overall improvement of cardiac haemodynamics. Newly transplanted cells should provide a three-dimensional structure that offers cell-to-cell electrical coupling and synchronised action and should ideally differentiate into cardiac cells at the target peri-infarction zone.

Our studies were based on a well-established mouse model of myocardial infarction and focused on molecular imaging techniques that allowed tracking of transplanted cells at various time points from short-term imaging (cell retention) to BLI-evaluated long-term maintenance cell biodistribution in engrafted organ. huSkM, MSCs alone or huSkM native or overexpressing Cx43 in combination with mesenchymal stem cells were administered. The positive effect of cell intervention in various variants was confirmed by functional MRI of cardiac haemodynamics.

Cellular therapy (intervention) was based on well-characterised human skeletal muscle stem progenitor cells (Figure 2a–e) and mesenchymal stem cells (Figure 3a–l) using well-described markers for both cell types [21,22,23]. Then, the cells were transduced with two different promoter reporter systems (MSCV-fluc-GFP and EF1-mkate-nanoluc), allowing for fluorescent and bioluminescent in vitro evaluation of generated cell suspensions (Figure 2f–h and Figure 3m–o), respectively. Reports from preclinical and clinical studies have shown that transplanted skeletal myogenic cells are able to differentiate into myotubes; however, unfortunately, there is no convincing data on their good electromechanical coupling with surrounding recipient cardiomyocytes, which in turn can lead to dangerous arrhythmias [24]. Two reasons for the lack of this functional connection are possible. On the one hand, the Cx43 expression observed in undifferentiated human myoblasts is strongly downregulated during their differentiation into myotubes. In addition, it has been reported that post-infarction cardiomyocytes significantly reduce their Cx43 expression. Overexpression of Cx43 in implanted myoblasts was one of the possible means to improve electrophysiological connections. Interestingly, the enhanced expression of Cx43 in cardiomyocytes was also obtained in in vitro culture in conditioned medium collected from mesenchymal stem cells or in vivo by co-transplantation with mesenchymal cells [2]. Additionally, earlier studies in animal models confirmed that cellular therapy based on MSCs alone was beneficial for cardiac function; however, this phenomenon requires further verification to determine whether this effect persists for a longer time [25].

Success of post-infarction cellular therapy depends on a variety of factors, such as time from MI, dose, method of cell delivery, their survival and proper engraftment at the target site. Therefore, we attempted to optimise each step of our therapeutic approach. According to du Pré, inflammatory conditions immediately after MI do not favour proper cell integration and immunological tolerance [25]. Therefore, in our mouse model, we set out the optimal time for cell delivery at 23–28 days post-infarction [10]. Intramyocardial injection targeting various sites in the vicinity of the post-infarction scar, starting from the centre of the injured myocardium to the border zone, is the first and most commonly used method of cell delivery. Even though targeting the centre of the damage can be beneficial, we delivered the cells (a total of 1.5 × 10^6^) into the border zone to achieve proper perfusion and maximise their survival and integration with neighbouring cardiomyocytes. This location was also recommended by other reports [26].

Intramyocardial injection itself is difficult to perform in small animals; thus, we have monitored cell delivery by SPECT/CT scans. This approach allowed us to verify and at the same time exclude animals with unsuccessful cell delivery. Interestingly, the highest percentage of short-term stem cell colonisation was observed in the case of huSkM overexpressing Cx43 combined with mesenchymal cells in post-infarction heart (Figure 5b, Supplementary Table S1), suggesting that connexin 43 expression and coadministration of MSCs that secreted paracrine factors may enhance skeletal myoblast retention in the target site. We have also observed that some stem cells were mechanically lost during the injection because they were squeezed out of the contracting heart (short-term visualisation of stem cell delivery). A similar effect was previously reported by Hudson et al. [27]. Escape of the cells from the injection site additionally contributes to the observed cell loss, which was seen during BLI follow-up in huSkM and huSkM + MSC in healthy heart (Figure 6a,b) and huSkM in post-infarction heart (Figure 7a). Human skeletal myogenic cells overexpressing Cx43 (huSkMCx43 + MSC) or not (huSkM + MSC) supported by mesenchymal stem cells were able to proliferate at the site of administration in the post-infarction heart (Figure 7b–d), while in the healthy heart, no change in signal intensity for huSkMCx43+ MSC was observed (Figure 6c,d). The total number of mesenchymal stem cells transplanted alone did not change significantly in healthy or post-infarction heart at the end of follow-up (Figure 6h and Figure 7h). It is important to note that a certain proportion of MSCs migrated out of the heart to the abdominal region, which indicated that during the first days after injection, the majority of these cells died or escaped; however, a small part of the population was able to adapt and proliferate if space was available (post-infarction heart). It is not known whether the cells delivered constantly migrate from the heart to the liver and/or proliferate in the liver. Some observations of that type were reported previously by Tran and Wang, who highlighted that although MSCs migrated out of the site of delivery, they did not deteriorate the functions of other organs and did not differentiate into the other cell types [28]. Similarly, results reported by Toma and colleagues showed that although the majority of MSCs at day 4 after the injection migrated to the other organs, while the remaining small proportion of the cells (0.44%) adapted over time and acquired morphological features of recipient cardiomyocytes [29].

Our final functional MRI experiment documented that post-MI intervention with human skeletal myogenic cells transplanted in combination with mesenchymal stem cells was the most beneficial because it improved left ventricular ejection fraction of the animal heart (Figure 9a). Additional extended observations are required to determine whether this positive effect would remain stable over longer time.

## 4. Materials and Methods

### 4.1. In Vitro Cell Culture

#### 4.1.1. husk

Human skeletal myoblasts were isolated from the remaining tissue fragments after the anterior cruciate ligament (ACL) surgical procedure. Written informed consents were obtained from the study participants for tissue donation and all other procedures, including protocols based on the recommendations for human tissue collection and were accepted by the Bioethics Committee at Poznan University of Medical Sciences, Poland, permission no. 818/13. We hereby confirm that all the methods used in the study were performed under the relevant guidelines and regulations. Cells were propagated, passaged and characterised in vitro (CD 56, anti-desmin, anti-heavy chain myosin, myotube formation test) as previously described [30]. The antibodies used for immunofluorescence and flow cytometry are listed in Supplementary Table S2. 

#### 4.1.2. Isolation and In Vitro Culture of Mesenchymal Stem Cells

MSCs were isolated from 50 mL of lipoaspirates obtained from the waste after liposuction. After washing with phosphate buffered saline (PBS), lipoaspirates were digested with collagenase solution (0.04%) at 37 °C for 45 min. Then, the cell suspensions were centrifuged, and the cell pellets were exposed to saponin (0.01%) to lyse residual erythrocytes. Subsequently, the suspensions were centrifuged, and purified lipoaspirate-derived mononuclear cells were transferred to minimum essential medium- alpha modification (α-MEM) culture medium (Institute of Immunology and Experimental Therapy, Polish Academy of Sciences, Wroclaw, Poland) supplemented with 10% fetal bovine serum (FBS) (Lonza, Basel, Switzerland), 1% penicillin/streptomycin (Lonza, Basel, Switzerland) and 1% UltraGlutamine (Lonza, Basel, Switzerland). Cells originating from the adipose tissue were maintained under the standard in vitro culture conditions in a humidified atmosphere of 5% CO_2_ at 37 °C, and after 3–4 days, the medium was removed together with residual nonadherent cells. Cells were monitored every day until they reached 90% confluence and were passaged using a trypsin/ethylenediamine tetraacetic acid (trypsin/EDTA) protocol at a 1:4 ratio. The mesenchymal properties of the cells were confirmed by flow cytometry and immunostaining (with antibodies listed in a Supplementary Table S2) as previously described by Kozlowska et al. [21]. Assessment of cell differentiation properties (according to the manufacturer’s instructions for a Stem Pro kit) was performed using the cell passage No 3 cells. Standard Oil Red O, Alizarin Red S and Alcian blue 8G staining was performed to visualise oil droplets in adipocyte culture, calcium deposits in osteoblasts and aggrecans in chondrocytes, respectively.

### 4.2. Construction of Lentiviral Plasmids 

#### 4.2.1. Lentiviral Packaging

MSCV-fluc-GFP (Supplementary Figure S2a) and EF1-mkate-nanoluc (Supplementary Figure S2b) vectors were produced by VectorBuilder and packed into lentiviral particles using a 2nd generation packaging system according to a previously described protocol [31].

#### 4.2.2. Lentiviral Transduction of huSkM and Mesenchymal Stem Cells

A day before the transduction, 3 × 10^6^ myoblasts or mesenchymal cells were seeded in 225 cm^2^ culture flasks. An hour before the transduction, the cells were incubated with polybrene (5 μg/mL). The proper amounts of MSCV-fluc-GFP lentiviral particles in the case of huSkM and EF1-mkate-nanoluc-PGK-puromycin lentiviral particles in the case of mesenchymal stem cells were resuspended in 15 mL of complete dulbecco’s modified eagle medium (DMEM) or α-MEM, respectively. After 24 h, 15 mL of fresh complete medium was added and incubated for 24 h. Then, the medium was replaced with fresh medium, and the cells were selected using 0.5 µg puromycin for 10 days. The transduction efficiency was evaluated using a fluorescence microscope (Leica DMi8) monitoring the emission at 485 nm or 610 nm for the detection of GFP and mkate, respectively. Untransduced huSkM and mesenchymal cells were used as the corresponding negative controls.

Bioluminescence intensity in huSkM transduced with MSCV-fluc-GFP and MSC transduced with EF1-mkate-nanoluc was measured using a luciferase assay system (Promega, Madison, WI, USA) and a Nano-Glo^®^ luciferase assay system, respectively. Samples (5 × 10^4^) of huSkM transduced with MSCV-fluc-GFP or 10^4^ MSCs transduced with EF1-mkate-nanoluc were washed in PBS and centrifuged (1500 rpm, 10 min). The cell pellet was lysed in 20 µL of lysis buffer, and 100 µL of appropriate substrate was added. Measurements were performed in triplicate using a GloMax luminometer (Promega, Madison, WI, USA). Untransduced cells were used as a negative control.

#### 4.2.3. Estimation of Insert Copy Number by Quantitative Real-Time Polymerase Chain Reaction (qPCR)

Genomic DNA was extracted using a whole blood extraction mini kit (Qiagen, Germantown, MD, USA). The DNA concentration was measured with a NanoDrop. qPCR was performed using primers specific for the MSCV-fluc-GFP reporter vector (forward: 5′-CGAGGCTACAAACGCTCTCA-3′ and reverse: 5′-CGAAGATGTTGGGGTGTTGC-3′) and for the EF1-mkate-nanoluc reporter vector (forward: 5′-CATGGTGAGCGAGCTGATTA-3′ and reverse: 5′-CGCCTTGATTCTCATGGTCT-3′). The insert copy numbers were normalised to *albumin* detected using specific primers (forward: 5′-GCTTATGGAGGGGTGTTTCA-3′ and reverse 5-’TGGAGACTGGCACACTTGAG-3′). The qPCR conditions were as follows: initial denaturation for 60 s at 95 °C and 50 cycles of denaturation for 20 s at 95 °C, annealing for 20 s at 60 °C and elongation for 20 s at 72 °C.

### 4.3. Connexin 43 Plasmid Construction and Cx43 Overexpression in huSkM

The huSkM transduced with MSCV-fluc-GFP reporter vector were transfected with pCiNeo plasmid (Promega, Fitchburg, WI, USA) containing the coding sequence for the Cx-43 gene (GJA1, NM_000165.3) using the previously described protocol [13].

### 4.4. Animal Model

The experimental procedures were approved by the Local Ethics Committee in Poznan, Poland, permission no. 16/2018. The experiments were performed on nonobese diabetic/severe combined immunodeficiency (NOD/SCID) mice purchased from Jackson Laboratory (Bar Harbor, ME, USA). Mice were kept in germ-free animal facilities with water and food *ad libitum.*

### 4.5. Induction and Evaluation of Myocardial Infarction

Myocardial infarction was induced as described previously [10]. Briefly, animals were anaesthetised with 2% isoflurane/98% oxygen gas mixture. The thorax was then opened through the fourth intercostal space, and left coronary artery ligation was performed. The muscle and skin were sutured. Successful MI induction was then confirmed by echocardiography and PET/CT analysis.

### 4.6. SPECT and CT Imaging

Molecular imaging was performed at the University of Warsaw, Biological and Chemical Research Centre. SPECT and CT scans were conducted using an Albira PET/SPECT/CT preclinical imaging system (Bruker, Billerica, MA, USA). The animals were divided into the corresponding combinations (Figure 1a,b) that were administered with various combinations of radiolabelled stem cells. The [^99m^Tc]Tc-HMPAO radioconjugate was synthesised by adding pertechnetate eluted from the ^99^Mo/^99m^Tc generator (Polatom, Otwock-Świerk, Poland) to a commercially available Ceretec kit (GE Healthcare, Chicago, IL, USA). The obtained product was added to the cell pellet and mixed using a rotating platform. The radioactivity of the cell suspension was measured with an Atomlab 500 dose calibrator (Biodex, Shirley, NY, USA). The cells were incubated for 30 min at 37 °C. Then, the labelled cells were washed twice in buffered physiological saline to remove the unbound compounds. The radioactivity was measured, and the radioefficiency of the process was calculated.

Mice underwent intubation and were kept under general anaesthesia for subsequent surgery (isoflurane/oxygen). The labelled cells were administered intramyocardially by three injections in a total volume of 30 µL. Then, the animals were weighed and transferred to a scanner chamber, where SPECT and CT imaging was performed. SPECT and CT imaging was repeated for all the mice after 23 ± 1 h. The obtained scans were merged using PMOD software version 3.3. An anatomical (CT) greyscale image and a physiological (SPECT) image on the cool-coloured scale were superimposed to determine the radioactivity accumulation sites originating from the labelled cells. SUVs (standardized uptake value) were calculated based on the manually outlined ROIs (regions of interest).

### 4.7. PET/CT

PET and CT scans were acquired using an Albira PET/SPECT/CT preclinical imaging system (Bruker, Billerica, MA, USA) as described previously by Fiedorowicz et al. [32]. PET and CT scans were merged using PMOD software version 4.02 and Fusion Tool module (PMOD Technologies LLC, Zurich, Switzerland).

### 4.8. Bioluminescent Imaging

Bioluminescent imaging was performed using an In vivo Imaging System (IVIS) (Perkin Elmer, Waltham, MA, USA). Briefly, animals were anaesthetised using a mixture of 2% isoflurane and 98% oxygen. To detect firefly luciferase expressed by huSkM MSCV-fluc-GFP, Xeno-light D-luciferin K^+^ salt substrate (Perkin Elmer, Waltham, MA, USA) was injected intraperitoneally (150 mg/kg body weight), and MSC EF1-mkate-nanoluc reporter was imaged using furimazine substrate (≈ 0.25 mg/kg, 40× dilution of Nano-Glo substrate, Promega, WI, USA) in 100 μL sterile PBS injected via the tail vein.

### 4.9. MRI Acquisition

Magnetic resonance imaging (MRI) was performed using a 7T Bruker Biospec scanner (70/30 USR, Bruker Biospin, Ettlingen, Germany) to assess functional cardiac parameters (ejection fraction and end-systolic and end-diastolic volumes for both ventricles). A set of coils included received only surface coil and transmitted cylindrical radiofrequency volume coil (10 mm and 8.6 cm inner diameter, respectively). Mice were placed in an MR-compatible animal bed and anaesthetized using 1.5% isoflurane in a mixture of oxygen and air. An animal monitoring system was used to monitor the respiration rate and heart-beat during the experiment (SA Instruments, Stony Brook, NY, USA). The imaging experiment started with pilot scans, and subsequent scans were used to establish the proper geometry for the 4-chamber view of the long axis of the heart. Based on the acquired 4-chamber view scan, the geometry for the set of short axis scans covering all ventricle volumes was established. IntraGateFLASH protocol was used for acquisition of short axis scans with parameters: echo time = 3 ms, repetition time = 10 ms, number of repetitions = 120, field of view = 25 mm × 25 mm, slice thickness = 0.9 mm, spatial resolution = 0.13 mm × 0.13 mm per pixel. Heartbeat cycle comprised 15 images. A gating system was used to improve image quality, and respiration and heart rate parameters were set manually.

### 4.10. MRI Data Analysis

The data were reconstructed in DICOM by the Bruker Paravision system. A set of short axis images represented the heartbeat cycle for each slice of the heart. Images showing end-systolic and end-diastolic moments of the heart cycle were defined. The inner edges of the left and right ventricles were manually outlined for each slice to calculate end-diastolic, end-systolic volume and ejection fractions for both ventricles. This analysis was performed using a custom-made plugin for Osirix (MRHeart by Dr. Konrad Werys).

### 4.11. Statistical Analysis

Statistical analysis was performed using GraphPad Prism software version 5.03 for Windows. The data are presented as the mean  ±  SD, * *p*  <  0.05, ** *p*  <  0.01, *** *p*  <  0.001, **** *p* < 0.0001.

## 5. Conclusions

The results presented herein demonstrated that cellular therapy based on the combination of huSkM with mesenchymal stem cells was the most beneficial variant for the mouse post-infarction heart. The differences, however, between native stem cell combinations or human skeletal myogenic cells overexpressing Cx43 delivered together with MSCs were not significant. This conclusion was drawn based on the overall results of molecular imaging of stem cell delivery assessed by SPECT, BLI and MRI. Our observations also indicated that mesenchymal stem cells supported the viability, proliferation and engraftment of human skeletal myogenic cells in post-infarction heart. Therefore, our subsequent studies will be focused on the molecular analysis of changes in critical signalling pathways in myocardium connected to electromechanical stability, resolution of fibrosis and improved angiogenesis to provide long-term follow-up basis for stem cell intervention based on cell variants selected in this study.

## Data Availability

The data presented in this study are available on request from the corresponding author.

## Supplementary Materials

The following are available online at https://www.mdpi.com/article/10.3390/ijms221910885/s1.

## Abbreviations

ACTB—actin protein, MSC—mesenchymal stem cells, huSkM—human skeletal myoblasts, IPSC—induced pluripotent stem cells, bFGF—basic fibroblast growth factor, BLI—bioluminescent imaging, G-CSF—granulocyte colony-stimulating factor, IGF-insulin like growth factor, EGF—epidermal growth factor, VEGF—vascular endothelial growth factor, MI—myocardial infarction, PGK—phosphoglycerate kinase, SPECT—Single-photon emission computed tomography, CT—computed tomography, MRI—magnetic resonance, IVIS—in vivo imaging system, LV—left ventricle, LV-EF—left ventricle ejection fraction, LV mass—left ventricle mass, RV-EF—right ventricle ejection fraction, RV mass—right ventricle mass, SUV—Standardized uptake value, DAPI—4′,6-diamidino-2-phenylindole, PET/CT—positron emission tomography/computed tomography, SPECT/CT—Single-photon emission computed tomography/computed tomography, MRI—magnetic resonance, [18F]-FDG PET/CT—18F-fluorodeoxyglucose (18F-FDG) positron emission tomography/computed tomography, [99mTc]Tc-HMPAO—Technetium-99m Hexamethylpropyleneamine Oxime, EF-1—elongation factor 1, mkate—red fluorescence, nanoluc—nanoluciferase, MSCV—murine stem cell virus, fluc—firefly luminescence, GFP—green fluorescent protein, huSkM+MSC—human skeletal myoblasts in combination with mesenchymal stem cells, huSkMCx43 + MSC—human skeletal myoblasts overexpressing Cx43 in combination with mesenchymal stem cells, q-PCR—quantitative polymerase chain reaction, PBS— phosphate buffered saline, FBS—fetal bovine serum, α-MEM—minimum essential medium- alpha modification, EDTA—ethylenediamine tetraacetic acid, DMEM—dulbecco’s modified eagle medium, NOD/SCID—nonobese diabetic/severe combined immunodeficiency.
