# Supplementary material for: Molecular Imaging of Human Skeletal Myoblasts (huSKM) in Mouse Post-Infarction Myocardium
Source: Int J Mol Sci. 2021 Oct 8;22(19):10885. doi: 10.3390/ijms221910885 (PMC8509689; doi:10.3390/ijms221910885)

Supplementary file

Molecular imaging of human skeletal myoblasts (huSkM) in mouse post-infarction myocardium.

Katarzyna Fiedorowicz , Weronika Wargocka-Matuszewska , Karolina A. Ambożkiewicz, Anna Rugowska, Łukasz Cheda, Michał Fiedorowicz, Agnieszka Zimna , Monika Drabik, Szymon Borkowski , Maciej Świątkiewicz, Piotr Bogorodzki , Paweł Grieb , Paulina Hamankiewicz , Tomasz Kolanowski , Natalia Rozwadowska, Urszula Kozłowska, Aleksandra Klimczak, Jerzy Kolasiński, Zbigniew Rogulski, Maciej Kurpisz

Corresponding author

Maciej Kurpisz MD, PhD

Institute of Human Genetics, Polish Academy of Sciences

Strzeszynska 32, 60-479 Poznan, Poland

E-mail: maciej.kurpisz@igcz.poznan.pl

Supplementary Figure S1 Flow cytometry phenotype characteristics of mesenchymal stem cells. Cells were positive for CD73 (a), CD90 (b) and CD 34 (c) markers and (d) negative for CD45 (haematopoietic marker).

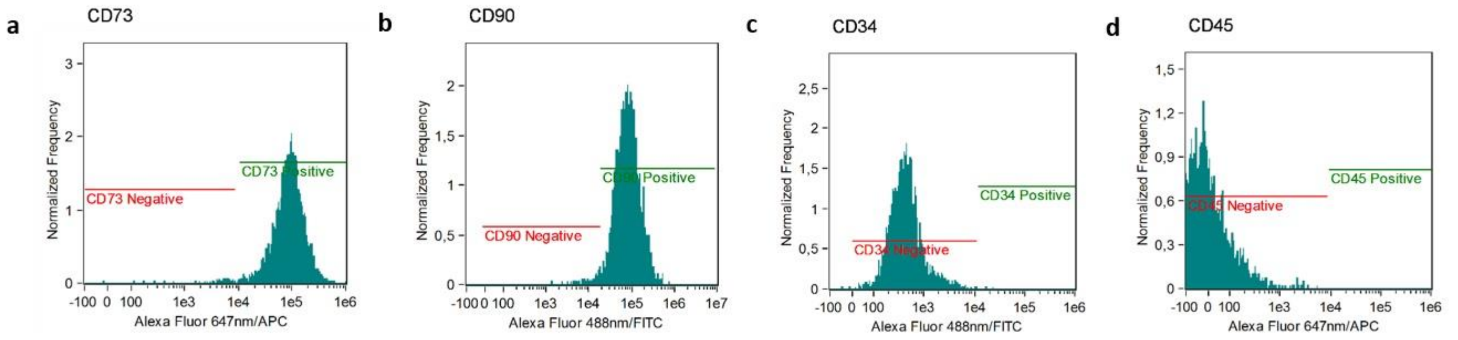

Alexa Fluor 647nm/APC

Population	Count	%Gated
MSC	3527	100
CD73 Negative & MSC	40	1,13
CD73 Positive & MSC	3479	98,6

Alexa Fluor 488nm/FITC

Population	Count	%Gated
MSC	3471	100
CD90 Negative & MSC	62	1,79
CD90 Positive & MSC	3398	97,9

Alexa Fluor 488nm/FITC

Population	Count	%Gated
MSC	3906	100
CD34 Positive & MSC	2	0,05
CD34 Negative & MSC	3900	99,8

Alexa Fluor 647nm/APC

Population	Count	%Gated
MSC	3906	100
CD45 Negative & MSC	2752	70,5
CD45 Positive & MSC	0	0

Supplementary Table S1. Comparison of the colonization percentage in the heart by using [99mTc]Tc-HMPAO -labelled stem cells. Results were obtained by comparing the ratio of the calculated SUVs from the first (0 min) and the second (23 +/- 1 h) SPECT/CT measurements, taking into account the half-life of [99mTc]Tc-HMPAO. The results are presented for each variant of the cells applied as the mean ± SD. The post-infarction group of mice was compared with the control group (healthy hearts: huSkM n=7, MSC n=8, huSkM+MSC n=4, huSkMCx43+MSC n=4; post-infarction hearts huSkM n=8, MSC n=3, huSkM+MSC n=8, huSkMCx43+MSC n=8. Data are presented as the mean ± SD, Mann-Whitney U test).

The percentage of colonization of [^{99m}Tc]Tc-HMPAO-labeled stem cells in the heart after 24h.

huSkM		MSC		huSkM+MSC		huSkMCx43+MSC	
control	post-infarction	control	post-infarction	control	post-infarction	control	post-infarction
47,23 ± 5,14	29,98 ± 8,22	19,24 ± 11,77	42,20 ± 15,98	39,29 ± 10,56	19,76 ± 8,40	23,03 ± 11,35	67,17 ± 9,30

Abbreviations: huSkM- human skeletal myoblasts, MSC- mesenchymal stem cells, huSkM+MSC- human skeletal myoblasts in combination with mesenchymal stem cells, huSkMCx43 + MSC- human skeletal myoblasts overexpressing Cx43 in combination with mesenchymal stem cells, SUV- standardized uptake value, SPECT/CT- single photon emission computed tomography/computed tomography, [99mTc]Tc-HMPAO- Technetium-99m Hexamethylpropyleneamine Oxime

Supplementary Table S2 List of antibodies used for immunofluorescence and flow cytometry.

Antibodies immunofluorescence	Manufacturer	Characteristics
mouse anti-desmin	Abcam Cambridge (UK)	Myogenic cells markers
mouse anti-heavy chain myosin		
mouse anti-CD 45	Abcam Cambridge (UK)	hematopoietic marker
rabbit anti-CD 73		mesenchymal cell markers
rabbit anti-CD 90		
mouse anti-CD 105		
anti-mouse Alexa Fluor 488	Abcam Cambridge (UK)	flurochrome conjugated secondary antibody
anti-rabbit Alexa Fluor 594		
**Antibodies – flow cytometry**	**Manufacturer**	**Characteristics**
CD 56 (FITC)	BD Pharminogen (USA)	Myogenic cell marker
CD 34 (FITC 581)		hematopoietic cell markers
CD 45 (APC HI30)		
CD 73 (APC AD2)		mesenchymal markers
CD 90 (FITC 5E10)		

Abbreviations: [99mTc]Tc-HMPAO - Technetium-99m Hexamethylpropyleneamine Oxime, SPECT/CT – single photon emission computed tomography/computed tomography.

Supplementary Figure S2
Map of lentiviral vectors. a. MSCV-fluc-GFP-puromycin lentiviral reporter vector (produced by VectorBuilder): Firefly luciferase and GFP expression are controlled by an MSCV constitutive promoter. The puromycin resistance cassette allows for the selection of the transgenes. b. EF1-mkate-nanoluc-PGK-puromycin lentiviral reporter vector (produced by VectorBuilder): expression of fluorescent mkate and bioluminescent nanoluc is controlled by constitutive elongation factor 1 (EF1) promoter, and the second constitutive phosphoglycerate kinase-1 (PGK) promoter allows the selection of transduced cells.

Abbreviations: MSCV- murine stem cell virus, PGK- *phosphoglycerate kinase*, fluc- firefly luminescence, GFP- green fluorescent protein mkate- red fluorescence, nanoluc- nanoluciferase.

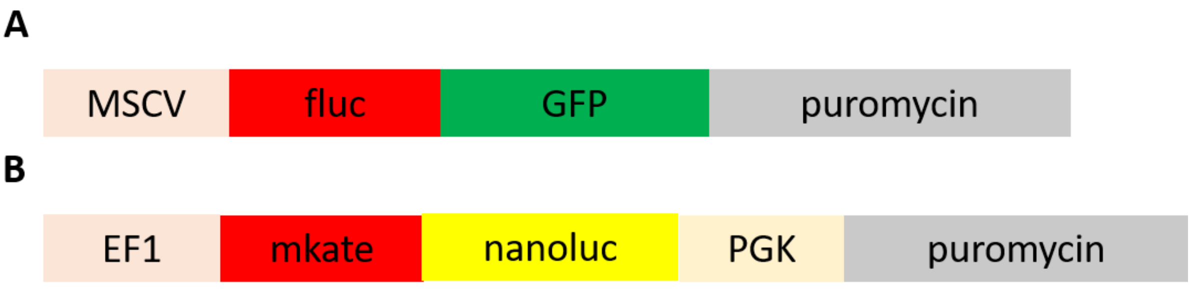

Supplement: Supplementary file 1 [file ijms-22-10885-s001.zip › ijms-1319874-supplementary.pdf]
